# EMERGENCY DEPARTMENT AND INPATIENT HOSPITAL HEALTHCARE UTILIZATION IN THE YEARS PRECEDING INCIDENT DEMENTIA

**DOI:** 10.1093/geroni/igac059.1624

**Published:** 2022-12-20

**Authors:** Raj Kumar, Katherine Ornstein, Evan Bollens-Lund, Jing Li, Ken Covinsky, Amy Kelley

**Affiliations:** Icahn School of Medicine at Mount Sinai, New York, New York, United States; Johns Hopkins, University, Baltimore, Maryland, United States; Icahn School of Medicine at Mount Sinai, New York, New York, United States; Cornell University, Ithaca, New York, United States; University of California, San Francisco, San Francisco, California, United States; Icahn School of Medicine at Mount Sinai, New York, New York, United States

## Abstract

There is evidence health utilization increases after incident dementia, particularly toward the end of life. However, less is known about utilization in the years before dementia. Our study objectives were to compare outpatient emergency department (ED) and inpatient hospital utilization in the six years preceding incident dementia compared to a reference group without dementia. We obtained data on n=5,547 Beneficiaries from the Health and Retirement Study-Medicare linked sample, and defined dementia using a validated algorithm. Those with (n=1,241) and without (n=4,306) dementia were balanced on confounders using inverse probability weighting applied to longitudinal Generalized Estimating Equation models. We found persons with dementia had greater odds of ED (OR=1.46, 95% CI: 1.21, 1.77) and inpatient hospital (OR=1.35, 95% CI: 1.12, 1.63) usage in the years preceding dementia compared to those without dementia across a comparable timespan. This study provides evidence to suggest greater healthcare burden may exist before manifestation of dementia.

